# Productivity Losses Due to Long-Term Back Problems in Working-Age Australians

**DOI:** 10.1001/jamanetworkopen.2025.27284

**Published:** 2025-08-22

**Authors:** Sean I. Docking, Ilana N. Ackerman, Rachelle Buchbinder, Ella Zomer, Danny Liew, Zanfina Ademi

**Affiliations:** 1 Musculoskeletal Health Unit, School of Public Health and Preventive Medicine, Monash University, Melbourne, Australia; 2 Wiser Health Care Unit, School of Public Health and Preventive Medicine, Monash University, Melbourne, Australia; 3 Health Economics Group, School of Public Health and Preventive Medicine, Monash University, Melbourne, Australia; 4 Diabetes Research Unit, School of Public Health and Preventive Medicine, Monash University, Melbourne, Australia; 5 Faculty of Health, Medicine and Behavioural Sciences, University of Queensland, Brisbane, Australia; 6 Health Economics and Policy Evaluation Research Group, Faculty of Pharmacy and Pharmaceutical Sciences, Monash University, Melbourne, Australia

## Abstract

**Importance:**

Long-term back problems impact an individual’s ability to participate in the workforce productively, potentially resulting in financial stress and furthering inequities. Estimates of future productivity losses could inform advocacy and policy making.

**Objective:**

To estimate the productivity losses of long-term back problems in working-age Australians (aged 15-64 years) over the next 10 years (2024-2033).

**Design, Setting, and Participants:**

This modeling study used a dynamic population-level model to simulate the population of working Australians with long-term back problems. Age- and sex-specific prevalence and workforce participation data were obtained from the 2022 National Health Survey. Excess all-cause mortality, absenteeism, and presenteeism data due to long-term back problems were derived from published sources.

**Main Outcomes and Measures:**

Primary outcomes were years of life lost, full-time equivalent workers lost, and productivity losses due to long-term back problems. Productivity losses were estimated as productivity-adjusted life-years and associated costs to Australia’s gross domestic product (GDP).

**Results:**

In 2024, 2 950 538 Australians had long-term back problems, which was projected to increase to 3 258 612 million by 2033. Long-term back problems resulted in an estimated loss of 3 394 255 productivity-adjusted life-years over the 10-year period, equating to a loss of more than 638 billion Australian dollars in Australia’s GDP. Reducing the relative prevalence and incidence of long-term back problems by 10% was estimated to result in a gain of 41.4 billion Australian dollars in GDP over the 10-year period.

**Conclusions and Relevance:**

In this modeling study estimating future productivity losses from long-term back problems, substantial economic gains could be achieved from reducing the prevalence and impact of the condition. This model highlights the need to assess the effectiveness of interventions on work-related outcomes.

## Key Points

**Question:**

What is the estimated cost of long-term back problems through early exit from the workforce, presenteeism, and absenteeism?

**Findings:**

In this modeling study using a dynamic population model, 3 258 612 Australians were projected to have long-term back problems by 2033, equating to an estimated loss of 638 billion Australian dollars in Australia’s gross domestic product over a 10-year period (2024-2033).

**Meaning:**

These findings suggest that action is needed to reduce long-term back problems by supporting workforce retention and improving care.

## Teaser

This modeling study uses a dynamic population model to estimate productivity losses associated with long-term back problems in working-age Australians from 2024 to 2033.

## Introduction

Back problems, also referred to as back pain, remains one of the leading causes of disability across all ages and sexes globally.^1,2^ In Australia, it was estimated to result in 240 000 years lived with disability in 2023, with the burden greatest in working-age Australians (aged 15-65 years).^3^ Beyond its health burden, the societal consequences of back problems are underappreciated and have not been well-quantified. Premature departure from the workforce and reduced productivity while working can create considerable financial stress and may disproportionally impact already disadvantaged communities.^4,5,6,7^

Productivity losses due to back problems represent a major economic burden to society. This population-level burden has been investigated in several countries using various approaches. A systematic review of cohort studies from 14 countries estimated that the prevalence of work absence due to back problems was 15.5%.^8^ By 2030, long-term back problems are projected to be the leading chronic condition associated with premature exit from the workforce. It would account for 21.4% of lost productive life-years for Australians aged 45 to 64 years,^9^ equating to a loss of 14.5 billion Australian dollars (A$) to Australia’s gross domestic product (GDP).^10^ This estimate may not fully capture productivity losses due to long-term back problems as this condition affects younger populations, and premature departure from the workforce is only 1 component of productivity loss.

Alongside established indicators, such as quality-adjusted life-years and disability-adjusted life-years, the productivity-adjusted life-year (PALY) is a novel metric to quantify disease burden and inform resource allocation decisions. As an advance over traditional productivity metrics, it combines data on mortality, absenteeism, presenteeism, and early workforce departure.^11^ Productivity-adjusted life-years have been used to estimate productivity losses due to a range of chronic conditions.^12,13,14,15^

The aim of our study was to quantify population-level productivity losses due to long-term back problems among working-age Australians. This study builds on previous research by, for the first time in our knowledge, measuring productivity losses across the full working-age population (aged 15-64 years) and by accounting for losses from absenteeism and presenteeism, as well as early workforce exit.

## Methods

In this modeling study, we developed a dynamic life table model with a 1-year cycle length to estimate current and future productivity losses due to long-term back problems in working-age Australians from 2024 to 2033. Ethics approval and informed consent were not sought as this study used only publicly available information. There is no analogous reporting guideline for the type of study reported here.

A dynamic model was used to account for future changes in the Australian population (migration, deaths, changes in prevalence of long-term back problems) and forecast the productivity impacts of long-term back problems in future years. Working age was defined as 15 to 64 years, consistent with Australian workforce and retirement definitions.^16^ Two sex- and age-specific life table models were constructed to estimate deaths, years of life lived, and productivity burden due to long-term back problems. One model estimated outcomes for people with long-term back problems, and a second model estimated outcomes for a comparable cohort with the assumption that this cohort did not have long-term back problems. Half-cycle corrections were made and an annual discount rate of 5% per annum applied to years of life lived, PALYs, and the GDP per Australian guidelines.^17^ The differences between the 2 modeled cohorts were assessed in terms of years of life lost, PALYs, and GDP loss. The model was built using Microsoft Excel, version 16.90.2 (Microsoft Corporation). This program is appropriate for the simple health economic modeling tasks required for this study and has been used for similar analyses.^18,19^ All model inputs, their sources, and parameter distributions are outlined in Table 1.

**Table 1.** Model Inputs, Their Sources, and Parameter Distribution

Input	Base case	Source	Parameter distribution
**Population**			
Australian population 2023	Age and sex specific	Australian Bureau of Statistics,^20^ 2024	NA
Population projections	Assumed mortality rates and net overseas migration, 2022 (base) to 2071	Australian Bureau of Statistics,^21^ 2024	NA
Prevalence of long-term back problems	Age and sex specific (eTable 1 in Supplement 1)	National Health Survey 2022,^23^ 2024	β
Excess mortality rate due to back problems, HR (95% CI)	1.17 (1.11-1.22)	Chen et al,^24^ 2021	Log-normal
**Productivity**			
Population workforce participation rate; financial year 2023-2024	Age and sex specific (eTable 2 in Supplement 1)^a^	Australian Bureau of Statistics,^25^ 2024	β
Workforce participation with long-term back problems, age-standardized rate (95% CI)	Employed, 0.727 (0.699-0.754); unemployed, 0.05 (0.032-0.069); not in labor force, 0.223 (0.197-0.248)	Australian Institute of Health and Welfare,^3^ 2024	Dirichlet
Workforce participation without long-term back problems, age-standardized rate (95% CI)	Employed, 0.772 (0.763-0.781); unemployed, 0.04 (0.034-0.045); not in labor force, 0.188 (0.18-0.197)	Australian Institute of Health and Welfare,^3^ 2024	Dirichlet
Average weekly total hours; financial year 2023-2024	Age and sex specific (eTable 3 in Supplement 1)	Australian Bureau of Statistics,^33^ 2024	γ
Absenteeism, percentage of a year	Back problems, 0.0696; no back problems, 0.0403	McDonald et al,^27^ 2011	β
Presenteeism, percentage of a year	Back problems, 0.276; no back problems, 0.143	McDonald et al,^27^ 2011	β
GDP per hours worked, 2024 Australian dollars	Projected based on data from 1975 to 2024 (eTable 4 in Supplement 1)	Australian Bureau of Statistics,^28^ 2024	NA

Abbreviations: GDP, gross domestic product; HR, hazard ratio; NA, not applicable.

^a^ Sensitivity analysis–assessed SE of 10% on employment rates.

### Model Population

Australian population size and structure data and projected mortality and net overseas migration rates were sourced from the Australian Bureau of Statistics to estimate the population size from 2024 to 2033 (Table 1).^20,21^ The medium series mortality rate and net overseas migration rates were used for the base case analysis. Age- and sex-specific prevalence rates for long-term back problems were obtained from the 2022 National Health Survey,^3^ which collects self-reported data from approximately 13 100 Australian households (eTable 1 in Supplement 1). As this dynamic model estimates outcomes for individual years of age, prevalence estimates by age decile were extrapolated to individual years of age using polynomial regression models that used the least-squares method in Microsoft Excel (eTable 1 in Supplement 1).^22^ Long-term back problems were classified as back pain or problems not elsewhere classified (disc disorders, sciatica, and curvature of the spine) that were current and had lasted, or was expected to last, 6 months or more (further description provided in the eMethods in Supplement 1). Incidence was calculated based on these prevalence estimates.^23^ We have made a conservative assumption that age-specific prevalence and incidence rates would remain unchanged over time based on data from previous National Health Surveys.^3^

National mortality rates for people with and without long-term back problems were estimated by adjusting Australian Bureau of Statistics all-cause mortality rates for the population with the increased risk of mortality associated with long-term back problems.^24^ Based on UK data, Chen et al^24^ reported that the hazard ratio for all-cause mortality among people with back pain was 1.17 (95% CI, 1.11-1.22) after adjusting for age, sex, ethnicity, and socioeconomic status (Table 1). It was assumed that net overseas migration was equally distributed across individuals with and without long-term back problems.

### Productivity Inputs

Age- and sex-specific proportions of Australians employed, unemployed (defined as those actively looking for work but not in the workforce), and not in the labor force (defined as those not actively looking for work or not available to start work) were obtained from the 2024 Australian Labour Force Survey (eTable 2 in Supplement 1).^25^ These rates were adjusted by the rate ratio of employed individuals with long-term back problems, compared with those without long-term back problems, based on an age-standardized workforce participation rate of 0.94.^3^ To calculate the number of full-time equivalent (FTE) workers with and without long-term back problems, we adjusted the number of individuals in the workforce by the average number of hours worked per week for each age and sex stratum (Table 1; eTable 3 in Supplement 1). The least-squares method was used to extrapolate age decile estimates to the aforementioned individual years of age.^22^ We defined a full-time workweek as 38 hours, consistent with the Australian Fair Work Act.^26^

Productivity indices were calculated to incorporate productivity loss attributed to both absenteeism and presenteeism, in which 0 was considered as completely unproductive and 1 as completely productive. No Australian-based studies were identified that measured productivity losses in individuals with and without long-term back problems. We therefore incorporated estimates from McDonald et al,^27^ who estimated work productivity in the 2008 US National Health and Wellness Survey (4920 participants with back problems and 25 948 without back problems). Absenteeism and presenteeism for individuals with and without long-term back problems are presented in Table 1. As measures of dispersion were not reported, we assumed an SE of 10%.

To estimate the number of PALYs for individuals with and without long-term back problems, the number of FTE workers was multiplied by the productivity indices. We also expressed PALYs in relation to Australia’s GDP. Data on the GDP per hour worked from the years 1975 to 2024 were obtained from the Australian Bureau of Statistics.^28^ We used a polynomial regression model using the least-squares method in Microsoft Excel to project GDP per hour worked beyond 2024, with the polynomial degree selected that best fit the data (*R*^2^ closest to 1) (eTable 4 in Supplement 1).^22^ This result was then converted into GDP per FTE, assuming a 38-hour workweek and 48 weeks in a work year. All costs are reported in 2024 Australian dollars (A$) and converted into US dollars ($0.66 for A$1^29^).

Years of life lost, PALYs lost, and GDP lost due to long-term back problems are reported for the overall population over the time horizon and for each age and sex stratum. The relative contribution to PALY losses was estimated for excess mortality, workforce participation, and productivity indices. To achieve this estimate, we reran the model 3 times, assuming that long-term back problems only impacted 1 of the excess mortality, workforce participation, and productivity indices. Relative contribution of each model input was calculated by dividing the PALY losses due to 1 model input by total PALY losses due to long-term back problems.

### Statistical Analysis

#### Scenario Analyses

We conducted several scenario analyses to assess the impact of certain assumptions made in the model. First, to highlight the potential benefits in reducing or preventing long-term back problems, we assumed hypothetical relative reductions of 10% and 25% in the prevalence and incidence of long-term back problems. Second, given uncertainty in the validity and applicability of productivity indices, we tested the model with productivity indices from 3 other sources (eTable 5 in Supplement 1).^30,31,32^ Third, to incorporate uncertainty in Australian population projections, we reran the model that assumed high net migration and life expectancy and in another model that assumed low net migration. Fourth, as a proportion of Australians continue to work beyond age 65 years, we reran the model including Australians aged 16 to 74 years. For those aged 65 to 74 years, the proportion of male individuals (19.1%) and female individuals (14.9%) in the workforce and average weekly hours (31 and 23 hours for male and female individuals, respectively) were sourced from the Australian Bureau of Statistics.^25,33^ Fifth, to test the assumption that GDP would continue to increase over time, we maintained the economic value of a PALY across the time horizon to 2024 levels (A$186 813 [$123 297] in GDP per FTE worker). Sixth, as per best practice recommendations, we used discount rates of 3% per annum and 0%.^17^ Seventh, as valuing productivity using GDP may overestimate the value of PALYs, we valued salary loss estimation using the human capital approach based on the average weekly earnings of full-time adult workers (eTable 6 in Supplement 1).

#### Sensitivity Analyses

One-way deterministic analyses were undertaken for all model inputs to ascertain the impact of input uncertainty and the robustness of model estimates. Probabilistic sensitivity analysis, running 10 000 Monte Carlo simulations, was performed to obtain 95% uncertainty intervals for PALYs and GDP.

## Results

Results of the base case model are presented in Table 2 and in eTable 7 in Supplement 1. A total of 2 950 538 Australians had long-term back problems in 2024, increasing to 3 258 612 by 2033. Slightly higher prevalence of long-term back problems was estimated in male compared with female individuals within our model (18.0% vs 15.5%) (eTable 8 in Supplement 1). However, when the margin of error for prevalence estimates was incorporated, the 95% CIs were similar (14.7%-21.2% vs 12.8%-18.2% for male and female individuals, respectively). Prevalence increased with age, from 7.6% (95% CI, 5.2%-9.9%) in individuals aged 15 to 24 years to 25.6% (95% CI, 22.2%-29.0%) in those aged 55 to 64 years (eTable 8 in Supplement 1).

**Table 2.** Projected Population and Discounted Years of Life Lived, PALYs, and Productivity Loss Expressed as GDP Over the 10-Year Time Horizon

Year	Projected population, No.		Years of life lived, No.		PALYs, No.		GDP, 2024 A$	
	With long-term back problems	Without long-term back problems	With long-term back problems	Lost due to long-term back problems^a^	With long-term back problems	Lost due to long-term back problems^a^	With long-term back problems	Lost due to long-term back problems^a^
2024	2 950 538	14 604 266	2 931 025	467	1 223 934	398 152	228 646 502 954	74 379 815 571
2025	2 988 067	14 814 413	2 827 907	1286	1 179 834	384 251	221 226 445 273	72 049 467 783
2026	3 022 309	15 006 424	2 725 794	1933	1 136 686	370 567	213 759 939 019	69 687 042 209
2027	3 055 627	15 191 229	2 625 174	2433	1 094 659	357 168	206 292 722 769	67 309 663 846
2028	3 088 084	15 367 949	2 527 223	2810	1 053 978	344 142	198 881 409 792	64 938 176 516
2029	3 121 098	15 537 720	2 432 528	3091	1 014 732	331 531	191 557 474 184	62 585 228 746
2030	3 155 715	15 702 832	2 341 927	3298	977 022	319 379	184 353 493 678	60 263 385 733
2031	3 191 205	15 866 409	2 255 319	3449	940 712	307 650	177 257 257 470	57 970 064 585
2032	3 225 598	16 013 706	2 171 573	3550	905 517	296 255	170 227 656 462	55 692 780 924
2033	3 258 612	16 151 069	2 089 890	3607	871 324	285 162	163 256 150 260	53 429 480 644
Total	31 056 852	154 256 017	24 928 360	25 925	10 398 397	3 394 255	1 955 459 051 860	638 305 106 555

Abbreviations: A$, Australian dollars; GDP, gross domestic product; PALY, productivity-adjusted life-year.

^a^ Calculated based on the difference in outcomes for people with long-term back problems compared with outcomes for a comparable cohort with respect to sex and age but with the assumption that this cohort did not have long-term back problems.

Few excess deaths were associated with long-term back problems (5.1 additional deaths per 100 000 population), resulting in minimal years of life lost (Table 2). The total number of PALYs lost by individuals with long-term back problems compared with those without over the 10-year time horizon was 3 394 255 (discounted PALY loss of 109 per 1000 individuals with long-term back problems), which equated to a loss of more than A$638 billion ($421 billion) to Australia’s GDP. For context, an average annual loss equates to 4.6% of Australia’s total GDP over the same 10-year period. A loss of A$20 553 ($13 565) to Australia’s GDP was estimated for each person with a long-term back problem. Examination of subgroup estimates showed that PALY losses were greatest in male individuals and individuals aged 45 to 54 years (eTable 8 in Supplement 1). Absenteeism and presenteeism due to long-term back problems contributed to most PALYs lost, accounting for 75.9% of losses (13.7% due to absenteeism and 62.2% due to presenteeism), followed by workforce departures (20.1%) and years of life lost (1.4%). The remaining PALYs lost (2.6%) was due to the interaction among excess mortality, loss to labor force, and/or productivity indices.

### Scenario Analyses

Under scenario 1, a 10% and 25% relative reduction in the prevalence of long-term back problems would translate to a gain of A$41.4 billion ($27.3 billion) and A$105.4 billion ($69.6 billion) to Australia’s GDP, respectively (eTable 9 in Supplement 1). Scenario 2 showed that incorporating productivity indices from different sources had a large impact on PALY losses. Using the productivity index reported by Gedin et al,^30^ which estimated that workers were absent from work 74 days of the year due to long-term back problems, resulted in a PALY loss of 4 848 549 and GDP loss of A$911.8 billion ($601.8 billion). Alternatively, using the productivity index reported by Allen et al,^31^ which estimated that the daily presenteeism due to long-term back problems was 16.7 minutes per day, resulted in a substantially lower PALY and GDP loss than observed in the base case (1 768 712 PALYs lost and A$332.6 billion [$219.5 billion], respectively). Similarly, lower productivity losses due to long-term back problems were observed when productivity indices from Kawai et al^32^ were used. For scenario 3, varying the assumptions underlying the Australian population projections had minimal impact on PALY losses and GDP losses (eTable 9 in Supplement 1).

For scenario 4, increasing the upper age limit to 74 years resulted in a further 443 589 Australians with long-term back problems in 2024, increasing to 825 520 by 2033. An additional 207 597 PALYs were lost due to long-term back problems over the 10-year time horizon, equating to A$39 billion ($25.7 billon) loss to GDP.

The PALY and GDP losses when the temporal GDP trend was removed (examined under scenario 5) and discounting rates were at 3% and 0% (examined under scenario 6) are presented in eTable 9 in Supplement 1. Assuming GDP gained per FTE worker remains at 2024 levels, GDP losses due to long-term back problems would be A$619.1 billion ($408.6 billion) compared with A$638.3 billion ($421.3 billion) in the base case. Approximately 4.2 million PALYs equating to A$791.1 billion ($522.1 billion) would be lost due to long-term back problems when no discount rate was applied. For scenario 7, valuing PALYs from a human capital approach based on average weekly salaries resulted in slightly smaller valuation of productivity losses due to long-term back problems (A$416.7 billion [$275.0 billion]) (eTable 10 in Supplement 1).

### Sensitivity Analyses

Results of the 1-way sensitivity analysis for selected variables are presented in the Figure. Uncertainty around productivity indices for both individuals with and without long-term back problems had a large impact on the estimates of PALYs lost. Uncertainty around employment rates for individuals with and without long-term back problems also substantially impacted the results. The model was robust to uncertainty for all other variables. Mean PALY losses due to long-term back problems over the 10-year time horizon were 3 430 045 (95% CI, 3 395 593-3 464 497) based on the probabilistic sensitivity analysis (eTable 11 in Supplement 1).

**Figure.**
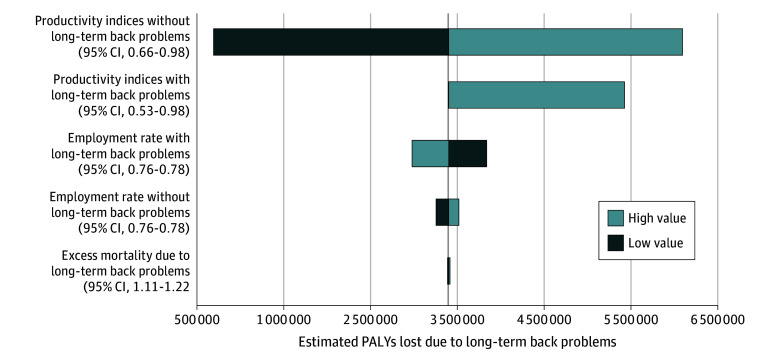
Tornado Plot Summarizing One-Way Deterministic Sensitivity Analysis for Selected Variables PALY indicates productivity-adjusted life-year.

## Discussion

In this modeling study, our dynamic life table model suggests that long-term back problems could result in the loss of 3.3 million PALYs over the next 10 years, equating to an average loss of A$63.8 billion ($42.1 billion) to Australia’s GDP each year. To put this finding in context, it is estimated that approximately A$112 billion ($73.9 billion) will be spent on health care for all health conditions in financial year 2024-2025.^34^ Reducing the prevalence of long-term back problems by 10% (or approximately 310 000 fewer cases each year) could result in 220 250 more PALYs and A$41.4 billion ($27.3 billion) of GDP gained over the 10-year period.

A previous study estimating productivity losses associated with back problems projected that in 2025, Australia’s GDP could face a loss of A$13.2 billion ($8.7 billion) among workers aged 45 to 64 years,^10^ which compared with our estimates for 2024, indicates a loss of A$33.1 billion ($21.9 billion) among workers aged 45 to 64 years. Previous studies frequently incorporate 1 component of productivity loss, namely early exit from the workforce.^10,35^ Our study highlights the importance of the PALY metric incorporating absenteeism and presenteeism, given that these were the largest contributors to productivity losses. Our findings highlight the need for health policies to focus not only on keeping individuals with long-term back problems in the workforce but also enabling them to adapt and work productively while in the workforce.^6^

Productivity-adjusted life-years have previously been estimated for a range of chronic conditions in various countries.^11,14,15,36^ Several studies in recent years have used dynamic structure to capture PALY loss in Australia for various conditions (type 2 diabetes, coronary heart disease, and kidney disease).^37,38,39,40^ Long-term back problems are one of the leading causes of reduced PALYs driven by their relatively high prevalence. For example, Savira et al^39^ estimated that 239 398 PALYs could be lost due to coronary heart disease from 2020 to 2029 compared with 3.4 million PALYs in our study. Our estimates highlight that modest reductions in the prevalence of long-term back conditions may yield a substantial return on investment at a societal level through a reduction in both direct and indirect costs.

A major way of addressing the burden of long-term back problems is to reduce the proportion of individuals who receive low-value or harmful care. Optimal treatment for people with acute, nonspecific, low-back pain focuses on the provision of adequate education around its favorable natural history, advice to stay active, and an emphasis on self-management.^41,42^ Remaining at work or returning to work as soon as possible with modifications, if needed, is also best practice. However, contrary to evidence-based guidelines,^41^ low-value care is prevalent.^43^ Examples of low-value care (diagnostic imaging in the absence of concerning features,^44,45^ use of opioid medications^46^) have been associated with longer absences from work.^47,48^ By contrast, high-value care could prevent chronicity, improve patient health outcomes, and result in productivity gains that benefit society.

### Strengths and Limitations

A key strength of this study is the use of a dynamic population model that accounts for new back problem cases and population changes, which is supported by national census and labor data to reduce bias. However, the study also has some limitations. A limitation around the validity of our estimates is the uncertainty in how long-term back problems impact absenteeism and presenteeism. Wide variation in productivity estimates were identified from various sources, and incorporating these estimates in our model had a large impact on the resulting PALY estimates.^27,30,31,32^ It is important to note that the estimated productivity losses associated with long-term back problems were not calculated as a direct consequence of the condition. For instance, approximately 75% of the excess mortality was mediated by lifestyle factors (physical activity, alcohol consumption, smoking status) and opioid use.^24^ We recognize that the direct productivity losses attributed to long-term back problems may be overestimated. Generalizability and applicability of these estimates to Australians with long-term back problems are unclear. Furthermore, the National Health Survey only captures self-reported long-term back problems that have lasted or are expected to last at least 6 months. Our data may be an underestimate of the full burden of back problems as acute and short-term disabilities were not captured.

## Conclusions

This modeling study highlights the substantial work-related burden of long-term back problems beyond their direct health care costs and quality-of-life impacts. Action is needed to reduce the prevalence and burden of long-term back problems, with a focus on better-quality care and supporting people of working age to remain in the workforce and working productively.

## Notes

Supplement 1. **eMethods.** Description of Long-Term Back Problems in the 2022 National Health Survey **eTable 1.** Prevalence of Back Problems by Age and Sex **eTable 2.** Australian Employment Rate by Age and Sex **eTable 3.** Average Weekly Hours and Proportion of Full-Time Workers by Age and Sex **eTable 4.** GDP Per Full-Time Equivalent Worker Estimated From 2024 to 2033 **eTable 5.** Absenteeism, Presenteeism, and Productivity Indices Used in Base Case and Scenario Analyses **eTable 6.** Average Weekly Salary Per Full-Time Equivalent Worker Estimated From 2024 to 2033 **eTable 7.** Projected Population and Discounted Year of Life Lived (YLL), Productivity-Adjusted Life-Years (PALYs), and Gross Domestic Product (GDP) for Those With and Without Long-Term Back Problems **eTable 8.** Projected Population and Discounted Year of Life Lived (YLL), Productivity-Adjusted Life-Years (PALYs), and Gross Domestic Product (GDP) by Sex and Age **eTable 9.** Projected Productivity-Adjusted Life-Years (PALYs) and Gross Domestic Product (GDP) for Scenario Analyses From 2024 to 2033 **eTable 10.** Estimated Productivity Losses Expressed as Lost Salary Due to Back Problems **eTable 11.** Results From Probabilistic Sensitivity Analysis Over the 10-Year Time Horizon
eReferences.

Supplement 2.
Data Sharing Statement
