# Supplementary material for: Productivity Losses Due to Long-Term Back Problems in Working-Age Australians
Source: JAMA Netw Open. 2025 Aug 22;8(8):e2527284. doi: 10.1001/jamanetworkopen.2025.27284 (PMC12374218; doi:10.1001/jamanetworkopen.2025.27284)
Supplement: Supplement 1. — eMethods. Description of Long-Term Back Problems in the 2022 National Health Survey eTable 1. Prevalence of Back Problems by Age and Sex eTable 2. Australian Employment Rate by Age and Sex eTable 3. Average Weekly Hours and Proportion of Full-Time Workers by Age and Sex eTable 4. GDP Per Full-Time Equivalent Worker Estimated From 2024 to 2033 eTable 5. Absenteeism, Presenteeism, and Productivity Indices Used in Base Case and Scenario Analyses eTable 6. Average Weekly Salary Per Full-Time Equivalent Worker Estimated From 2024 to 2033 eTable 7. Projected Population and Discounted Year of Life Lived (YLL), Productivity-Adjusted Life-Years (PALYs), and Gross Domestic Product (GDP) for Those With and Without Long-Term Back Problems eTable 8. Projected Population and Discounted Year of Life Lived (YLL), Productivity-Adjusted Life-Years (PALYs), and Gross Domestic Product (GDP) by Sex and Age eTable 9. Projected Productivity-Adjusted Life-Years (PALYs) and Gross Domestic Product (GDP) for Scenario Analyses From 2024 to 2033 eTable 10. Estimated Productivity Losses Expressed as Lost Salary Due to Back Problems eTable 11. Results From Probabilistic Sensitivity Analysis Over the 10-Year Time Horizon eReferences. [file jamanetwopen-e2527284-s001.pdf]

Supplemental Online Content

Docking SI, Ackerman I, Buchbinder R, Zomer E, Liew D, Ademi Z. Productivity losses due to long-term back problems in working-age Australians. *JAMA Netw Open*. 2025;8(8):e2527284. doi:10.1001/jamanetworkopen.2025.27284

eMethods. Description of Long-Term Back Problems in the 2022 National Health Survey

eTable 1. Prevalence of Back Problems by Age and Sex

eTable 2. Australian Employment Rate by Age and Sex

eTable 3. Average Weekly Hours and Proportion of Full-Time Workers by Age and Sex

eTable 4. GDP Per Full-Time Equivalent Worker Estimated From 2024 to 2033

eTable 5. Absenteeism, Presenteeism, and Productivity Indices Used in Base Case and Scenario Analyses

eTable 6. Average Weekly Salary Per Full-Time Equivalent Worker Estimated From 2024 to 2033

eTable 7. Projected Population and Discounted Year of Life Lived (YLL), Productivity-Adjusted Life-Years (PALYs), and Gross Domestic Product (GDP) for Those With and Without Long-Term Back Problems

eTable 8. Projected Population and Discounted Year of Life Lived (YLL), Productivity-Adjusted Life-Years (PALYs), and Gross Domestic Product (GDP) by Sex and Age

eTable 9. Projected Productivity-Adjusted Life-Years (PALYs) and Gross Domestic Product (GDP) for Scenario Analyses From 2024 to 2033

eTable 10. Estimated Productivity Losses Expressed as Lost Salary Due to Back Problems

eTable 11. Results From Probabilistic Sensitivity Analysis Over the 10-Year Time Horizon

eReferences

This supplemental material has been provided by the authors to give readers additional information about their work.

© 2025 Docking SI et al. *JAMA Network Open*.

eMethods. Description of Long-Term Back Problems in the 2022 National Health Survey

A Users' Guide and Questionnaire has not been published for the 2022 National Health Survey; however, the Australian Bureau of Statistics states that "the 2022 National Health Survey is considered to be comparable to the 2017-18 National Health Survey and previous cycles"[1].

The specific questions and available response options relevant to back problems in the Other Long Term Conditions module are as follows:

> *Earlier I asked you some questions about whether [you were/[first name] was] restricted in [your/his/her] day to day activities because of any medical conditions [you/[first name]] had, and I asked you about specific medical conditions.*
>
> *I would now like to ask you about any other long term health conditions that have lasted, or are expected to last, for 6 months or more.*
>
> *[Do you/Does [first name]] have any of these conditions? (Yes/No)*
>
> *Which of these [do you/does [first name]] have? (Response options include 'Back - slipped disc or other disc problems' and 'Back pain or other back problems')*
>
> *Please provide more information about [your/[first name]'s] back pain or back problem (60 characters for noting by the interviewer)*

Self-reported conditions are classified according to ICD-10 codes. Self-reported conditions that are classified as 'Dorsopathies' [back problems] include the sub-classifications of: 'Sciatica', 'Disc disorders', 'Curvature of the spine' or 'Back pain or problems not elsewhere classified'.

© 2025 Docking SI et al. *JAMA Network Open*.

eTable 1. Prevalence of Back Problems by AGE and sex

Age range, years	Age midpoint, years	Prevalence (95% CI)	Prevalence (95% CI)
		Females	Males
15 to 24	19.5	0.075 (0.048 to 0.102)	0.066 (0.044 to 0.088)
25 to 34	29.5	0.136 (0.108 to 0.164)	0.117 (0.09 to 0.144)
35 to 44	39.5	0.176 (0.15 to 0.202)	0.204 (0.168 to 0.24)
45 to 54	49.5	0.165 (0.141 to 0.189)	0.238 (0.198 to 0.278)
55 to 64	59.5	0.24 (0.208 to 0.272)	0.298 (0.258 to 0.338)
65 to 74	69.5	0.249 (0.215 to 0.283)	0.298 (0.26 to 0.336)
75+	75	0.273 (0.229 to 0.317)	0.282 (0.234 to 0.33)

Source: [2]

Sex-specific regression models

Females:

$y = 0.0000001444x^3 - 0.0000290744x^3 + 0.0049845749x^2 + 0x$

$R^2 = 0.994$

Males:

$y = -0.0000020446x^4 + 0.0002036006x^3 + 0.0000091078x^2 + 0x$

$R^2 = 0.999$

Note: x denotes year of age; y denotes the estimated prevalence.

Age-specific incidence rate formula

$$Incidence\ rate_{age} = \frac{prevalence\ rate_{age+1} - prevalence\ rate_{age}}{1 - prevalence\ rate_{age}}$$

© 2025 Docking SI et al. *JAMA Network Open*.

eTable 2. Australian Employment Rate by Age and Sex

Age range, years	Age midpoint, years	Proportion (95% CI)	Proportion (95% CI)
		Females	Males
15-24	19.5	0.651 (0.523 to 0.779)	0.632 (0.508 to 0.756)
25-34	29.5	0.799 (0.642 to 0.956)	0.871 (0.700 to 1)
35-44	39.5	0.805 (0.647 to 0.963)	0.889 (0.715 to 1)
45-54	49.5	0.797 (0.641 to 0.953)	0.862 (0.693 to 1)
55-64	59.5	0.621 (0.499 to 0.743)	0.725 (0.583 to 0.867)

Source: [3]

Regression model

Females:

$y = -0.0000011780x^5 + 0.0001839285x^4 - 0.0107189027x^3 + 0.2764006334x^2 - 1.8564281310x$

$R^2 = 1.000$

Males:

$y = -0.0000010042x^5 + 0.0001679083x^4 - 0.0106212146x^3 + 0.2993106854x^2 - 2.2656683023x$

$R^2 = 1.000$

Note: x denotes year of age; y denotes the estimated proportion.

© 2025 Docking SI et al. *JAMA Network Open*.

eTable 3. Average Weekly Hours and Proportion of Full-Time Workers by Age and Sex

Age range, years	Age midpoint, years	Average weekly hours (95% CI)	Proportion of full-time workers (95% CI)	Average weekly hours (95% CI)	Proportion of full-time workers (95% CI)
		**Females**		**Males**	
**15-17**	16	10.2 (9.2 to 11.2)	0.268 (0.243 to 0.294)	15.4 (13.0 to 17.8)	0.405 (0.343 to 0.467)
**18-20**	19	19.3 (18.1 to 20.5)	0.508 (0.477 to 0.539)	26.4 (24.8 to 28.0)	0.695 (0.653 to 0.736)
**21-24**	22.5	25.2 (24.22 to 26.2)	0.663 (0.637 to 0.689)	30.4 (29.2 to 31.6)	0.800 (0.769 to 0.831)
**25-34**	29.5	29.9 (29.5 to 30.3)	0.787 (0.777 to 0.797)	35.3 (34.7 to 35.9)	0.929 (0.913 to 0.944)
**35-44**	39.5	29.4 (29.0 to 29.8)	0.774 (0.763 to 0.784)	37.0 (36.4 to 37.6)	0.974 (0.958 to 0.989)
**45-54**	49.5	30.0 (29.6 to 30.4)	0.789 (0.779 to 0.800)	37.6 (37.2 to 38.0)	0.989 (0.979 to 1)
**55-64**	59.5	29.5 (28.9 to 30.1)	0.776 (0.761 to 0.792)	36.2 (35.6 to 36.8)	0.953 (0.937 to 0.968)

Source: [3]

Proportion of full-time equivalent workers was calculated by dividing average weekly hours with the maximum number of work hours in a week (38 hrs)

Regression model

Females:

$y = -0.0000014745x^5 + 0.000252698x^4 - 0.0158494x^3 + 0.431007256x^2 - 3.506525975x$

$R^2 = 0.999$

Males:

$y = -0.0000016036x^5 + 0.000266523x^4 - 0.016280124x^3 + 0.435352748x^2 - 3.360217153x$

$R^2 = 0.989$

Note: x denotes year of age; y denotes the estimated proportion of full-time workers.

© 2025 Docking SI et al. *JAMA Network Open*.

eTable 4. GDP Per Full-Time Equivalent Worker Estimated From 2024 to 2033

Year	GDP per FTE (AUD$ 2024)
2024	186812.7
2025	187506.5
2026	188055.4
2027	188453.8
2028	188696.0
2029	188776.4
2030	188689.2
2031	188428.8
2032	187989.5
2033	187365.7

Source: [4]

Regression model

$y = -0.946052211x^3 + 5674.848919x^2 - 11344299.79x + 7557763058$

$R^2 = 0.993$

Note: x denotes year; y denotes the estimated GDP per FTE worker.

© 2025 Docking SI et al. *JAMA Network Open*.

eTable 5. Absenteeism, Presenteeism, and Productivity Indices Used in Base Case and Scenario Analyses

	Absenteeism	Presenteeism	Productivity indices
	With back problems		
Base case	0.070	0.276	0.683
Scenario 1	0.203	0.276	0.521
Scenario 2	0.070	0.035	0.900
Scenario 3	0.015	0.043	0.898
	Without back problems		
Base case	0.040	0.143	0.817
Scenario 1	0.079	0.143	0.777
Scenario 2	0.040	0.012	0.947
Scenario 3	0.006	0.043	0.951

Base case: Both absenteeism and presenteeism used from McDonald et al [5]
Scenario 1: Absenteeism utilised from Gedin et al [6] and presenteeism from McDonald et al [5]
Scenario 2: Absenteeism utilised from McDonald et al [5] and presenteeism from Allen et al [7]
Scenario 3: Both absenteeism and presenteeism used from Kawai et al [8]

© 2025 Docking SI et al. *JAMA Network Open*.

eTable 6. Average Weekly Salary Per Full-Time Equivalent Worker Estimated From 2024 to 2033

Year	Weekly salary per FTE (AUD$ 2024)
2024	1993.63
2025	2085.00
2026	2190.46
2027	2311.57
2028	2449.90
2029	2607.02
2030	2784.48
2031	2983.86
2032	3206.72
2033	3454.62

Source: [9]

Regression model

$y = 0.260751748x^3 - 1577.021553x^2 - 3179300.029x - 2136527871$

$R^2 = 1$

Note: x denotes year; *y* denotes the estimated average weekly salary per FTE worker.

© 2025 Docking SI et al. *JAMA Network Open*.

eTable 7. Projected Population and Discounted Year of Life Lived (YLL), Productivity-Adjusted Life Years (PALYs), and Gross Domestic Product (GDP) for Those With and Without Long-Term Back Problems

Year	Projected population		YLL		PALYs		GDP (in AUD$ 2024)	
	With long term back problems	Without long-term back problems	With long-term back problems	Without long-term back problems	With long-term back problems	Without long-term back problems	With long-term back problems	Without long-term back problems
2024	2,950,538	14,604,266	2,931,025	14,495,307	1,223,934	7,493,947	228,646,502,954	1,399,964,505,105
2025	2,988,067	14,814,413	2,827,907	14,008,894	1,179,834	7,229,777	221,226,445,273	1,355,630,087,587
2026	3,022,309	15,006,424	2,725,794	13,524,189	1,136,686	6,971,223	213,759,939,019	1,310,976,120,378
2027	3,055,627	15,191,229	2,625,174	13,042,934	1,094,659	6,718,778	206,292,722,769	1,266,179,333,175
2028	3,088,084	15,367,949	2,527,223	12,570,556	1,053,978	6,473,235	198,881,409,792	1,221,473,907,985
2029	3,121,098	15,537,720	2,432,528	12,107,700	1,014,732	6,234,748	191,557,474,184	1,176,973,280,127
2030	3,155,715	15,702,832	2,341,927	11,656,091	977,022	6,003,399	184,353,493,678	1,132,776,739,050
2031	3,191,205	15,866,409	2,255,319	11,217,835	940,712	5,778,729	177,257,257,470	1,088,879,046,416
2032	3,225,598	16,013,706	2,171,573	10,788,858	905,517	5,559,616	170,227,656,462	1,045,149,633,676
2033	3,258,612	16,151,069	2,089,890	10,366,850	871,324	5,346,079	163,256,150,260	1,001,671,635,717
Total	31,056,852	154,256,017	24,928,360	123,779,214	10,398,397	63,809,531	1,955,459,051,860	11,999,674,289,216

© 2025 Docking SI et al. *JAMA Network Open*.

eTable 8. Projected Population and Discounted Year of Life Lived (YLL), Productivity-Adjusted Life Years (PALYs), and Gross Domestic Product (GDP) by Sex and Age

	Projected population		YLL		PALYs		GDP (AUD$ 2024)	
	With back problems[a]	Without back problems[a]	With back problems[b]	Lost YLL due to back problems[b]	With back problems	Lost PALYs due to back problems	With back problems	Lost GDP due to back problems
**Base case**	**3,258,612**	**16,151,069**	**24,928,360**	**25,925**	**10,398,397**	**3,394,255**	**1,955,459,051,860**	**638,305,106,555**
**Sex**								
**Females**	1,520,201	8,233,537	11,557,250	8,133	3,976,148	1,295,977	1,207,720,876,934	394,588,364,674
**Males**	1,738,411	7,917,532	13,371,110	17,791	6,422,249	2,098,277	747,738,174,926	394,588,364,674
**Age, years**								
**15-24**	290,890	3,551,445	2,232,432	0	557,708	181,312	104,883,192,208	34,097,628,327
**25-34**	511,065	3,575,189	4,049,113	0	1,795,946	583,864	337,722,706,218	109,793,981,969
**35-44**	752,792	3,528,032	5,757,269	1,100	2,690,002	875,230	505,882,636,426	164,596,146,763
**45-54**	849,679	3,004,035	6,228,117	7,020	2,948,439	963,086	554,451,754,628	181,108,434,833
**55-64**	854,187	2,492,369	6,661,430	17,805	2,406,301	790,763	452,518,762,380	148,708,914,664

[a] Projected population estimated for 2033

[b] Cumulative YLL, PALYs, and GDP over the 10-year time horizon

© 2025 Docking SI et al. *JAMA Network Open*.

eTable 9. Projected Productivity-Adjusted Life Years (PALYs) and Gross Domestic Product (GDP) for Scenario Analyses From 2024 to 2023

	PALYs		GDP (AUD$ 2024)	
	**Total**	**PALY gained/lost**	**Total**	**GDP gained/lost**
**Hypothetical reduction in prevalence of back problems**				
**Base case**	74,207,929	-	13,955,133,341,076	-
**10% reduction**	74,428,180	220,251	13,996,552,808,889	41,419,467,813
**25% reduction**	74,768,341	560,412	14,060,522,100,122	105,388,759,046
**Productivity indices**				
**Base case**	74,207,929	-	13,955,133,341,076	-
**Scenario 1**	69,032,981	- 5,174,948	12,981,962,233,612	-973,171,107,463
**Scenario 2**	88,252,223	14,044,294	16,596,224,478,229	2,641,091,137,154
**Scenario 3**	88,592,399	14,384,470	16,660,196,123,754	2,641,091,137,154
**Population projections**				
**Base case**	74,207,929	-	13,955,133,341,076	-
**High assumption**	74,979,188	771,259	14,100,290,915,087	145,157,574,011
**Low assumption**	73,613,935	- 593,993	13,843,343,855,731	-111,789,485,345
**GDP trend removed**				
**Base case**	74,207,929	-	13,955,133,341,076	-
**GDP trend removed**	74,207,929	0	13,535,526,219,434	-419,607,121,642
**Discount rate**				
**Base case (5%)**	74,207,929	-	13,955,133,341,076	-
**3%**	80,558,961	6,351,033	15,150,509,259,164	54,721,776,339
**0%**	91,940,263	17,732,334	17,292,496,221,116	152,783,762,916

© 2025 Docking SI et al. *JAMA Network Open*.

eTable 10. Estimated Productivity Losses Expressed as Lost Salary Due to Back Problems

Year	With back problems	Lost salary due to back problems[a]
2024	117,123,759,815.44	38,100,926,721
2025	118,077,829,359.25	38,455,821,825
2026	119,513,448,536.35	38,962,112,223
2027	121,458,359,815.66	39,629,712,870
2028	123,942,851,530.19	40,469,457,548
2029	126,980,434,495.70	41,486,763,035
2030	130,584,067,171.88	42,686,676,848
2031	134,733,816,114.82	44,063,234,044
2032	139,379,330,962.32	45,600,243,262
2033	144,484,234,363.00	47,285,922,098
**Total**	**1,276,278,132,164.62**	**416,740,870,473**

[a] Calculated based on the difference in outcomes for people with long-term outcomes compared to outcomes for comparable cohort with respect to sex and age but with the assumption that this cohort did not have long-term back problems.

eTable 11. Results From Probabilistic Sensitivity Analysis Over the 10-Year Time Horizon

	Projected population		YLL		PALYs		GDP (AUD$ 2024)	
	With back problems	Without back problems	With back problems	Lost YLL due to back problems	With back problems	Lost PALYs due to back problems	With back problems	Lost GDP due to back problems
**Mean**	**31,070,897**	**154,242,211**	**24,939,657**	**26,014**	**10,378,578**	**3,430,045**	**1,951,732,020,232**	**645,035,660,199**
**Lower limit (95% CI)**	31,053,339	154,224,617	24,925,560	25,913	10,355,789	3,395,593	1,947,446,384,313	638,556,754,042
**Upper limit (95% CI)**	31,088,456	154,259,806	24,953,754	26,116	10,401,368	3,464,497	1,956,017,656,151	651,514,566,356

© 2025 Docking SI et al. *JAMA Network Open*.

eReferences

1. Australian Bureau of Statistics. National Health Survey methodology, 2022. May 10, 2024. Accessed December 12, 2024. https://www.abs.gov.au/methodologies/national-health-survey-methodology/2022

2. Australian Institute of Health and Welfare. Chronic musculoskeletal conditions : Back problems. Australian Institute of Health and Welfare. June 17, 2024. Accessed October 9, 2024. https://www.aihw.gov.au/reports/chronic-musculoskeletal-conditions/back-problems

3. Australian Bureau of Statistics. Labour Force, Australia, Detailed, July 2024. October 24, 2024. Accessed November 7, 2024. https://www.abs.gov.au/statistics/labour/employment-and-unemployment/labour-force-australia-detailed/jul-2024

4. Australian Bureau of Statistics. Australian System of National Accounts, 2023-24 financial year. October 25, 2024. Accessed November 7, 2024. https://www.abs.gov.au/statistics/economy/national-accounts/australian-system-national-accounts/latest-release

5. McDonald M, DiBonaventura M daCosta, Ullman S. Musculoskeletal pain in the workforce: the effects of back, arthritis, and fibromyalgia pain on quality of life and work productivity. *J Occup Environ Med*. 2011;53(7):765-770. doi:10.1097/JOM.0b013e318222af81

6. Gedin F, Alexanderson K, Zethraeus N, Karampampa K. Productivity losses among people with back pain and among population-based references: a register-based study in Sweden. *BMJ Open*. 2020;10(8):e036638. doi:10.1136/bmjopen-2019-036638

7. Allen D, Hines EW, Pazdernik V, Konecny LT, Breitenbach E. Four-year review of presenteeism data among employees of a large United States health care system: a retrospective prevalence study. *Hum Resour Health*. 2018;16(1):59. doi:10.1186/s12960-018-0321-9

8. Kawai K, Kawai AT, Wollan P, Yawn BP. Adverse impacts of chronic pain on health-related quality of life, work productivity, depression and anxiety in a community-based study. *Fam Pract*. 2017;34(6):656-661. doi:10.1093/fampra/cmx034

9. Australian Bureau of Statistics. Average Weekly Earnings, Australia, May 2024. August 15, 2024. Accessed November 19, 2024. https://www.abs.gov.au/statistics/labour/earnings-and-working-conditions/average-weekly-earnings-australia/latest-release

© 2025 Docking SI et al. *JAMA Network Open*.
