# Supplementary material for: Productivity Losses Due to Long-Term Back Problems in Working-Age Australians
Source: JAMA Netw Open. 2025 Aug 22;8(8):e2527284. doi: 10.1001/jamanetworkopen.2025.27284 (PMC12374218; doi:10.1001/jamanetworkopen.2025.27284)
Supplement: Supplement 2. — Data Sharing Statement [file jamanetwopen-e2527284-s002.pdf]

Data Sharing Statement

Docking. Productivity Losses Due to Long-Term Back Problems in Working-Age Australians. *JAMA Netw Open*. Published August 15, 2025. doi:10.1001/jamanetworkopen.2025.27284

Data
Data available: Yes
Data types: Other (please specify)
Additional Information: The model and data supporting the findings of this study are available upon reasonable request from the corresponding author.
How to access data: The model and data supporting the findings of this study are available upon reasonable request from the corresponding author.
When available: With publication

Supporting Documents
Document types: Statistical/analytic code
How to access documents: Through request to corresponding author (sean.docking@monash.edu)
When available: With publication

Additional Information
Who can access the data: Available upone reasonable requests
Types of analyses: Specific purpose to estimate the productivity losses related to back problems
Mechanisms of data availability: With investigator support
